# Sensitivity Assessment of Macroscopic Mechanical Responses of Cemented Sand and Gravel to Mesoscopic Parameters Using an Improved MULTIMOORA Method

**DOI:** 10.3390/ma19183917

**Published:** 2026-09-15

**Authors:** Zhangyu Shi, Fan Li, Yanan Zhang

**Affiliations:** 1 College of Civil and Transportation Engineering, Hohai University, Nanjing 210098, China; 251304020021@hhu.edu.cn; 2 Hubei Key Laboratory of Water Engineering Materials and Application Technology, China Three Gorges Corporation, Wuhan 430010, China; 3 College of Water Resources and Hydropower, Hohai University, Nanjing 210098, China

**Keywords:** cemented sand and gravel, random aggregate finite element model, mesoscopic parameters, relative sensitivity, MULTIMOORA, multi-criteria decision making, parameter prioritization

## Abstract

The macroscopic mechanical responses of cemented sand and gravel (CSG) are jointly affected by the mesoscopic properties of the aggregate, mortar matrix, and interfacial transition zone, while different parameters exhibit distinct magnitudes and directions of influence on strength, stiffness, and failure deformation. To establish a unified parameter priority across multiple macroscopic responses, a two-dimensional random aggregate finite element model was developed, and the elastic moduli and tensile strengths of the aggregate, mortar matrix, and interface were selected as six mesoscopic parameters. Bidirectional perturbations of ±5% and ±10% were introduced around the baseline state, and dimensionless local sensitivity coefficients were calculated using a central finite-difference formulation. Five statistically independent random aggregate realizations were further considered to evaluate the influence of mesostructural variability. The Ordered Weighted Averaging (OWA) operator and entropy weighting method were used to determine subjective and objective criterion weights, respectively, and a MULTIMOORA-based multi-response parameter-prioritization framework was established using the ratio system, reference point method, and modified multiplicative form. Across the five random realizations, the mean sensitivities of the mortar elastic modulus to the macroscopic elastic modulus and failure displacement are 0.5158 and 0.2664, respectively, while those of the interfacial tensile strength to compressive strength and failure displacement are 0.1550 and 0.4512, respectively. The ±5% and ±10% perturbations yield consistent parameter hierarchies, indicating stable local sensitivity results within the investigated neighborhood of the baseline state. Under the combined OWA–entropy weighting scheme, the multi-response parameter priority is ranked as mortar elastic modulus, interfacial tensile strength, aggregate elastic modulus, interfacial elastic modulus, mortar tensile strength, and aggregate tensile strength. The ratio system and modified multiplicative form yield identical rankings, and both show a Spearman rank correlation coefficient of 0.9429 with the reference point method. Weight-scenario analysis further shows that the mortar elastic modulus and interfacial tensile strength consistently remain the two highest-priority parameters, although their internal order varies with the criterion weights. The resulting ranking therefore represents a model- and evaluation-objective-dependent multi-response parameter priority and provides a quantitative basis for mesoscopic parameter screening and subsequent calibration of CSG numerical models.

## 1. Introduction

Cemented sand and gravel (CSG) materials are produced by mixing natural sand and gravel with a small amount of cementitious materials and water. Owing to their advantages of local material availability, low cementitious material consumption, and high construction efficiency, CSG materials have considerable application potential in hydraulic structures and dam engineering [1]. Because of the wide particle-size range of aggregates, the pronounced differences in the mechanical properties of individual constituents, and the random distribution of interfaces, CSG materials exhibit significant heterogeneity under loading. Their macroscopic strength, stiffness, and deformation characteristics are therefore not governed by any single material parameter but arise from the coordinated interaction among the aggregate skeleton, mortar matrix, and interfacial transition zones.

Previous studies on the relationships between macroscopic and mesoscopic parameters of composite materials have employed finite element methods, particle flow simulations, and machine-learning techniques for parameter calibration and influence analysis. Random aggregate finite element models can explicitly represent aggregate morphology and interfacial distributions, particle flow models are well suited to investigating force-chain development and crack evolution, and data-driven approaches such as random forests and neural networks can characterize nonlinear mappings between multiple input parameters and macroscopic responses. Huang Xiao [2] combined the random forest method with the controlled-variable approach to analyze the effects of macroscopic and mesoscopic parameters in the rolling-resistance linear contact model and the rolling-resistance Hertz–Mindlin model for sandy soil. The relative importance of the parameters was determined, and the applicability of the random forest method to discrete element parameter calibration was verified. Li Keyu et al. [3] established a numerical model using PFC and conducted a large number of unconsolidated, undrained triaxial simulations on red clay. By varying parameters such as the friction coefficient and bond strength, they examined the effects of mesoscopic parameters on the stress–strain response. Taking Beishan granite as an example, Xu Jinming et al. [4] identified the types and spatial locations of mesoscopic constituents using laboratory test images and threshold segmentation. The constituent particles and cementing materials were represented by discs and parallel bonds, respectively, to establish a particle flow model incorporating the actual distribution of mesoscopic constituents. On this basis, they proposed a method for investigating the relationships between macroscopic and mesoscopic mechanical properties and for calibrating mesoscopic mechanical parameters. Huang Hu et al. [5] performed extensive compression tests on cemented sand and gravel materials using the particle-based discrete element method and investigated the correlations between mesoscopic parameters and macroscopic mechanical properties. Benvenuti et al. [6] proposed an artificial neural network-based parameter identification method for DEM simulations of soils, which links macroscopic experimental results with microscopic numerical parameters and facilitates the characterization and prediction of material properties.

From the perspective of sensitivity analysis, existing methods can generally be divided into local or one-at-a-time perturbation analyses around a baseline state and global sensitivity analyses over the entire parameter space. The Morris screening method can identify influential input variables with a limited number of model evaluations, whereas Sobol and variance-based methods further quantify the contributions of individual parameters and parameter interactions to output variance. Response-surface and surrogate-model-based sensitivity methods provide another computationally efficient strategy by approximating the mapping between input parameters and model responses. These approaches can capture nonlinear parameter–response relationships while reducing the number of expensive numerical simulations, although their reliability depends on the parameter range, sampling strategy, and predictive accuracy of the surrogate model. In the present study, local finite-difference analysis is adopted to quantify response-specific sensitivities in the neighborhood of the baseline state, whereas MULTIMOORA is subsequently used for a different purpose, namely, to integrate the sensitivities associated with multiple macroscopic responses into an engineering-oriented parameter priority. Therefore, sensitivity calculation and multi-response prioritization are treated as two consecutive but conceptually distinct stages of the proposed framework. Global sensitivity methods are well suited to capturing nonlinear effects and interactions over broad parameter ranges, but their computational cost increases substantially for models with expensive individual evaluations, such as random-aggregate finite element models. Although one-at-a-time relative perturbation cannot replace global sensitivity analysis, it enables a direct comparison of changes in macroscopic responses while keeping all other parameters and model geometries unchanged and therefore remains useful for preliminary parameter screening and prioritization before calibration. Another issue that has received less attention is that the overall performance of a parameter scheme is not equivalent to the sensitivity of macroscopic responses to parameter variations [7,8,9]. Compressive strength, elastic modulus, and failure displacement differ in physical meaning, units, and response direction; therefore, directly ranking raw macroscopic responses may confound performance assessment with parameter sensitivity [10,11,12]. Moreover, even when the sensitivity of a mesoscopic parameter to each macroscopic response is obtained separately, a unified priority across multiple response criteria is still required.

MULTIMOORA integrates three multi-criteria aggregation mechanisms—the ratio system, reference point approach, and multiplicative form—and evaluates the same decision alternative from the perspectives of linear compensation, deviations from the ideal state, and multidimensional joint responses. Accordingly, this study clearly distinguishes response sensitivity calculations from multi-response parameter prioritization. First, dimensionless finite-perturbation relative sensitivity is used to quantify the magnitude of the effect of each mesoscopic parameter on an individual macroscopic response while retaining the response direction. The sensitivity magnitudes are then used to construct a multi-attribute decision matrix. Combined subjective–objective weights are obtained using the OWA–entropy weighting scheme, and MULTIMOORA is subsequently applied to determine the multi-response parameter priority. MULTIMOORA is not intended to replace global sensitivity methods such as Morris or Sobol analysis; rather, it is used to integrate response-specific sensitivities into an engineering priority ranking and to examine the dependence of the ranking on aggregation mechanisms and weighting scenarios.

## 2. Improved MULTIMOORA-Based Local Sensitivity Evaluation Method

### 2.1. Evaluation Parameters and Local Sensitivity Coefficients

Let the set of mesoscopic parameters of cemented sand and gravel be defined as $A=\left\{A_{1},A_{2},\ldots ,A_{m}\right\}$, and the set of macroscopic response indicators as $C=\left\{C_{1},C_{2},\ldots ,C_{n}\right\}$. The elastic modulus and tensile strength of the aggregate, mortar matrix, and interface are selected as six mesoscopic parameters, while compressive strength, macroscopic elastic modulus, and failure displacement are taken as the three macroscopic response indicators.

To quantify the local response of the j-th macroscopic indicator to the i-th mesoscopic parameter in the neighborhood of the baseline state, a dimensionless central finite-difference sensitivity coefficient is defined as [13]:

(1) $$R_{ij}^{\left(h\right)}=\frac{y_{j}\left[\left(1+h\right)x_{i0}\right]-y_{j}\left[\left(1-h\right)x_{i0}\right]}{2hy_{0j}}$$

where $x_{i0}$ is the baseline value of the ith mesoscopic parameter; h is the prescribed relative perturbation amplitude; $y_{0j}$ is the baseline value of the jth macroscopic response; and $y_{j}\left[\left(1+h\right)x_{i0}\right]$ and $y_{j}\left[\left(1-h\right)x_{i0}\right]$ are the corresponding responses obtained after positive and negative perturbations of parameter i, respectively.

A positive $R_{ij}^{\left(h\right)}$ indicates that the macroscopic response increases with the parameter in the neighborhood of the baseline state, whereas a negative value indicates a decreasing response. For subsequent multi-response parameter prioritization, the sensitivity magnitude is defined as:

(2) $$S_{ij}^{\left(h\right)}=\left|R_{ij}^{\left(h\right)}\right|$$

A larger $S_{ij}^{\left(h\right)}$ indicates a stronger local response to the corresponding parameter variation. It should be emphasized that $S_{ij}^{\left(h\right)}$ quantifies only the magnitude of the response variation and does not retain its direction. Therefore, a high sensitivity does not necessarily imply that increasing the corresponding parameter improves material performance. The physical meaning of each sensitivity should be interpreted together with the sign of $R_{ij}^{\left(h\right)}$ and the original macroscopic response.

In the present study, h=0.05 and h=0.10 are adopted to obtain local sensitivities using ±5% and ±10% bidirectional perturbations, respectively. A comparison of the two perturbation amplitudes is used to assess the stability of the local sensitivity estimates near the baseline state.

In addition to the local perturbations, the original +100% one-at-a-time perturbation cases are retained as finite-amplitude comparison cases. These results are used only to examine the response tendency over a substantially larger parameter variation and are not treated as the sole basis for strict local sensitivity assessment.

### 2.2. OWA–Entropy Combined Weighting Method

Compressive strength, elastic response, and failure deformation differ in their importance for engineering evaluation. To reduce the influence of individual extreme ratings on subjective weights, the OWA operator is used to aggregate expert scores [14]. Let $N_{e}$ denote the total number of experts and k denote the ordered position of an expert score. For the jth criterion, the $N_{e}$ independent scores are arranged in descending order as $b_{1j}\geq b_{2j}\geq \cdots \geq b_{N_{e}j}$, and the corresponding OWA positional weights are calculated as:

(3) $$\omega_{k}=\frac{\left(\begin{array}{c} N_{e}-1\\ k=1 \end{array}\right)}{2^{N_{e}-1}},\;\;\;\;k=1,2,\ldots ,N_{e}$$

When $N_{e}=6$, the resulting positional-weight vector is 0.03125, 0.15625, 0.31250, 0.31250, 0.15625, and 0.03125. The OWA aggregate score of the j-th criterion is then calculated as:

(4) $$B_{j}=\sum_{k=1}^{N_{e}}\omega_{k}b_{kj}$$

The subjective weights are further normalized as:

(5) $$W_{j}^{s}=\frac{B_{j}}{\sum_{j=1}^{n}B_{j}}$$

The objective weights are determined from the dispersion of information in the sensitivity matrix. First, the following quantity is calculated:

(6) $$p_{ij}=\frac{S_{ij}}{\sum_{i=1}^{m}S_{ij}}$$

The entropy value is calculated as [15]:

(7) $$e_{j}=-\frac{1}{\ln m}\sum_{i=1}^{m}p_{ij}\ln p_{ij}$$

When $p_{ij}=0$, the following limit relation is applied:

(8) $$\underset{p\to 0}{\lim\;}p\ln p=0$$

When $p_{ij}=0$, $p_{ij}\ln p_{ij}=0$ is set to 0.

The divergence coefficient and objective weight are then calculated as:

(9) $$d_{j}=1-e_{j}$$

(10) $$W_{j}^{o}=\frac{d_{j}}{\sum_{j=1}^{n}d_{j}}$$

The combined weights are obtained through multiplicative normalization [15]:

(11) $$W_{j}=\frac{W_{j}^{s}W_{j}^{o}}{\sum_{j=1}^{n}W_{j}^{s}W_{j}^{o}}$$

### 2.3. Improved MULTIMOORA-Based Comprehensive Ranking

MULTIMOORA is a multi-criteria decision-making method. MULTIMOORA is employed in this study as a multi-response aggregation framework after the response-specific sensitivity coefficients have been obtained. Its purpose is not to replace the sensitivity calculation itself, but to examine the comprehensive priority of mesoscopic parameters when several macroscopic responses are considered simultaneously [16]. Before applying MULTIMOORA, the sensitivity-magnitude matrix is normalized column-wise using vector normalization:

(12) $$X_{ij}^{*}=\frac{S_{ij}}{\sqrt{\sum_{i=1}^{m}S_{ij}^{2}}}$$

(1) Ratio system

Since all three criteria represent sensitivity magnitudes, they are treated as benefit-type criteria. The overall evaluation value of the ratio system is calculated as:

(13) $$Y_{i}=\sum_{j=1}^{n}W_{j}X_{ij}^{*}$$

A larger $Y_{i}$ indicates a higher overall sensitivity of the corresponding parameter.

(2) Reference Point Method

The reference point method takes the maximum normalized value of each criterion rⱼ=maxᵢ(xᵢⱼ*) as the ideal point and uses the Chebyshev distance to calculate the maximum weighted deviation:

(14) $$D_{i}={max}_{j}\left\{W_{j}\left|r_{j}-X_{ij}^{*}\right|\right\}$$

A smaller $D_{i}$ indicates that the parameter is closer to the ideal sensitivity state across all responses.

(3) Modified multiplicative form

In the conventional multiplicative form, the product becomes zero when $X_{ij}^{*}=0$, which may eliminate the contribution of the remaining nonzero responses. To retain zero-sensitivity responses while preserving the monotonicity of the evaluation function, a shifted weighted geometric form is adopted:

(15) $$Uᵢ=\prod_{j=1}^{n}{\left(1+X_{ij}^{*}\right)}^{W_{j}}$$

Adding 1 shifts the criterion domain from the nonnegative range to values no smaller than 1, while preserving the monotonic ordering of each criterion; that is, a larger $X_{ij}^{*}$ still yields a larger contribution to the evaluation. This transformation also prevents the entire product from collapsing because of a zero entry. However, the shift changes the numerical discrimination among different sensitivity levels. Therefore, the final ranking is not determined by $U_{i}$ alone, but by jointly considering the ratio system, reference point method, and modified multiplicative form.

The three aggregation mechanisms provide complementary perspectives on parameter priority. The ratio system is a fully compensatory aggregation mechanism, in which a high sensitivity with respect to one response may compensate for a relatively low sensitivity with respect to another response. The reference point method emphasizes the maximum weighted deviation from the ideal response state and therefore gives greater attention to the least favorable response dimension. The multiplicative form evaluates the joint contribution of different response criteria and reduces the dominance of a single linearly compensated criterion. Accordingly, MULTIMOORA is not introduced to artificially alter the ranking obtained from a simple weighted sum. Instead, it is used to examine the extent to which the identified parameter priority depends on the selected aggregation mechanism. Agreement among the different sub-methods is therefore interpreted as evidence of cross-method consistency rather than as proof of algorithmic superiority.

The three sub-methods yield separate rankings. If one parameter ranks higher than another across all three methods, it is considered dominant. When no complete dominance relationship can be established, the parameter with the smaller sum of ranks is given higher priority. If the rank sums are equal, the parameter with the larger ratio-system value $Y_{i}$ is ranked higher.

### 2.4. Cross-Method Consistency and Weight Robustness

To assess the stability of different ranking methods, the Spearman rank correlation coefficient is used to quantify the degree of agreement between any two rankings:

(16) $$\rho =1-\frac{6\sum_{i=1}^{m}d_{i}^{2}}{m\left(m^{2}-1\right)}$$

where $d_{i}$ denotes the difference in rank of the same parameter between the two ranking methods. A value of ρ closer to 1 indicates a higher degree of agreement between the two rankings.

The directly weighted sensitivity is also calculated as:

(17) $$C_{i}=\sum_{j=1}^{n}W_{j}S_{ij}$$

Because the directly weighted method and MULTIMOORA are based on the same sensitivity matrix, agreement between their rankings is used only to assess internal consistency among different aggregation approaches and is not regarded as an independent validation of the physical accuracy of the numerical model or the true parameter ranking. To evaluate the dependence of the results on criterion weights, four weighting scenarios are further considered: equal weights, OWA-based subjective weights, entropy-based objective weights, and combined OWA–entropy weights. The resulting parameter priorities are then compared across these scenarios.

## 3. Random Aggregate Numerical Model and Parameter Scheme

### 3.1. Finite Element Model

CSG consists of natural gravel aggregates, a mortar matrix, and aggregate–mortar interfacial transition zones. Differences in the mechanical properties and spatial distributions of these constituents lead to pronounced mesoscopic heterogeneity [17,18]. To represent this feature, a two-dimensional random polygonal aggregate finite element model with dimensions of 100 mm × 100 mm was established in ANSYS (ANSYS APDL 17.0) The material was divided into three phases: aggregate, mortar matrix, and interface.

**Figure 1:**
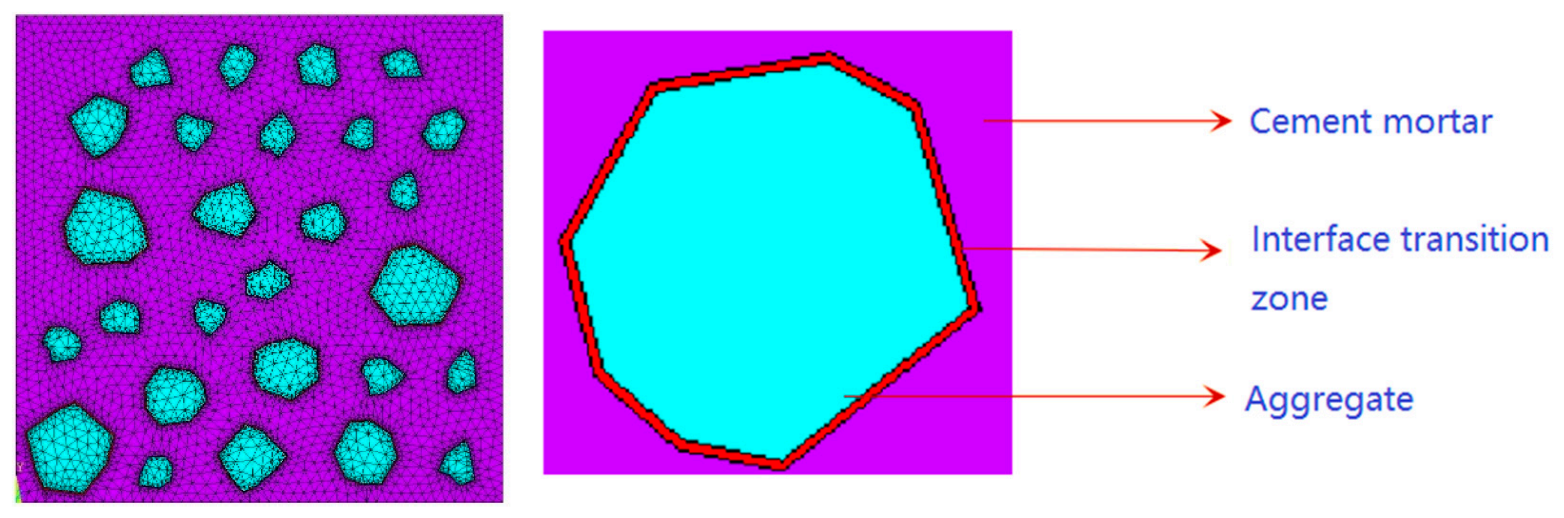
Mesoscopic random aggregate model of CSG.

Based on the two-grade aggregate gradation of CSG, aggregate locations were randomly generated using the Monte Carlo method, while the Walraven approach was adopted to convert the three-dimensional aggregate gradation into the corresponding two-dimensional particle-size and number distributions. Aggregates were sequentially placed from larger to smaller sizes while satisfying the prescribed minimum spacing and boundary constraints. Convex polygonal aggregates were then generated using randomly distributed vertices, resulting in a two-dimensional mesostructure with random aggregate locations, sizes, and shapes [19,20]. The resulting random polygonal aggregate distribution is shown in Figure 1.

The model was discretized using an automatic triangular mesh, with distinct material properties assigned to the aggregate, mortar matrix, and interface phases. Because the aggregate–mortar interface is a region of pronounced material-property discontinuity and local stress concentration, the mesh was refined near the interface, while a relatively coarser mesh was adopted within the aggregates to balance response resolution and computational efficiency. All three phases were modeled using linear-elastic constitutive relations, with their mechanical differences represented by the elastic modulus, Poisson’s ratio, and tensile strength. A two-dimensional plane stress assumption was adopted, and the model was discretized using eight-node PLANE82 structural solid elements. A phase-dependent meshing strategy was employed, with characteristic element sizes of approximately 7 mm in the aggregate phase and 2 mm in the mortar matrix, while the aggregate–mortar interface was locally refined to 0.5 mm. The interface was explicitly represented as a finite-width equivalent interfacial layer surrounding each polygonal aggregate. Its inner boundary was generated by scaling the corresponding aggregate contour to 95% of the local radial dimension; thus, the interface thickness varied proportionally with the local aggregate size rather than being prescribed as a constant physical ITZ thickness. The Poisson’s ratios of the aggregate, mortar matrix, and interface were 0.16, 0.22, and 0.20, respectively.

To ensure a consistent basis for comparison among different mesoscopic parameter cases, the specimen dimensions, aggregate geometry, interface distribution, mesh, and boundary conditions were kept unchanged within each random realization, and only the target mesoscopic parameter was varied [21,22]. This treatment isolates the effect of parameter variation from changes in mesostructural geometry. To further evaluate the influence of random aggregate distribution, five statistically independent random aggregate realizations were additionally considered.

### 3.2. Failure Criterion and Numerical Implementation

During uniaxial compression, differences in stiffness and strength among the aggregate, mortar matrix, and interface can induce local tensile stress concentrations and initiate cracking. Based on previous random aggregate finite element studies, the maximum tensile stress criterion was adopted to identify mesoscopic element failure. An element was considered to have failed when its maximum principal tensile stress reached the tensile strength of the corresponding material phase:

(18) $$\sigma_{1}\geq f_{t}$$

where $\sigma_{1}$ is the maximum principal tensile stress of the element, and $f_{t}$ is the tensile strength of the corresponding material phase. Once the failure criterion is satisfied, the load-carrying capacity of the failed element is reduced using the ANSYS element birth-and-death technique to represent local damage and crack propagation. Uniaxial compression was applied through displacement-controlled loading. The bottom boundary was constrained in both the horizontal and vertical directions ($U_{x}=U_{y}=0$), while the top boundary was constrained horizontally ($U_{x}=0$) and subjected to a prescribed downward displacement. An initial vertical displacement of 0.01 mm was applied, followed by increments of 0.006 mm per load step. Each load step was divided into five substeps, with automatic time stepping adopted to facilitate convergence. Once the maximum tensile stress criterion was satisfied, the corresponding element was deactivated using the ANSYS element birth-and-death procedure. The stiffness of a deactivated element was reduced to $1.0\times 10^{-6}$ of its original value, corresponding to the default ANSYS deactivation setting. The same loading, boundary, and element-deactivation settings were used for all simulation cases.

For all parameter cases, the specimen geometry, random aggregate distribution, mesh, boundary constraints, and loading conditions were kept unchanged, with only the target mesoscopic parameter varied. During uniaxial compression, the macroscopic load, deformation, and element stress responses were extracted for subsequent sensitivity analyses of compressive strength, macroscopic elastic modulus, and failure displacement.

### 3.3. Baseline Mesoscopic Parameters and Multilevel Perturbation Scheme

The baseline mesoscopic parameters provide the reference state for the subsequent local sensitivity analysis. The elastic moduli and tensile strengths of the aggregate, mortar matrix, and interface were not independently recalibrated in the present study. Instead, the equivalent mesoscopic parameter set reported in reference [18] was adopted because the same type of random aggregate finite element framework and numerical implementation were used in that study.

Relevant experimental comparisons and macro–meso parameter calibration had already been conducted in reference [18]. In that study, the equivalent mesoscopic parameters of the aggregate, mortar matrix, and interface were determined through a macro–meso inversion procedure by matching the numerical macroscopic mechanical responses with the corresponding experimental responses of CSG. The present study directly adopts this calibrated parameter system and numerical framework as the baseline for sensitivity analysis, rather than performing an independent recalibration. Accordingly, the parameters adopted here should be interpreted as equivalent mesoscopic finite element parameters associated with the specific discretization scale, phase representation, interface treatment, and inversion procedure, rather than as intrinsic elastic moduli or tensile strengths measured directly from homogeneous aggregate or mortar specimens. This distinction is particularly important for values such as the 500 MPa aggregate elastic modulus, which should not be directly compared with the conventional bulk elastic modulus of mineral aggregates.

The present study focuses on the changes in macroscopic responses induced by parameter variations within this established equivalent parameter system rather than on identifying the intrinsic material constants of each constituent phase. The parameter values listed in Table 1 were therefore adopted as the baseline state for the subsequent one-at-a-time sensitivity analysis.

A multilevel one-at-a-time perturbation scheme is adopted around the baseline state. For each mesoscopic parameter, relative perturbations of −10%, −5%, +5%, and +10% are applied while all other parameters are kept at their baseline values. The ±5% and ±10% cases are used to calculate local central finite-difference sensitivities and to examine their stability with respect to perturbation amplitude. The original +100% perturbation cases are retained only as finite-amplitude comparison cases to investigate whether the response tendency observed near the baseline state remains representative over a substantially larger parameter variation. See Table 2 below for details.

### 3.4. Random Mesostructural Realizations

Because the sensitivity results of a random aggregate model may depend on the specific mesostructural realization, five statistically independent random aggregate configurations are generated using different random seeds. The specimen dimensions, aggregate grading, aggregate area fraction, polygon-generation rules, material parameters, boundary conditions, and mesh-generation strategy are kept statistically identical, while the aggregate locations, polygonal shapes, and interface spatial distributions vary among the realizations.

For each random realization, the baseline state and the ±5% perturbation cases of the six mesoscopic parameters are simulated. The resulting local sensitivity coefficients are used to calculate the mean value, standard deviation, coefficient of variation, and ranking variability of each parameter. This procedure is used to distinguish the effect of parameter variation from the variability introduced by the random mesostructure.

### 3.5. Numerical Loading and Extraction of Macroscopic Responses 

**Figure 2:**
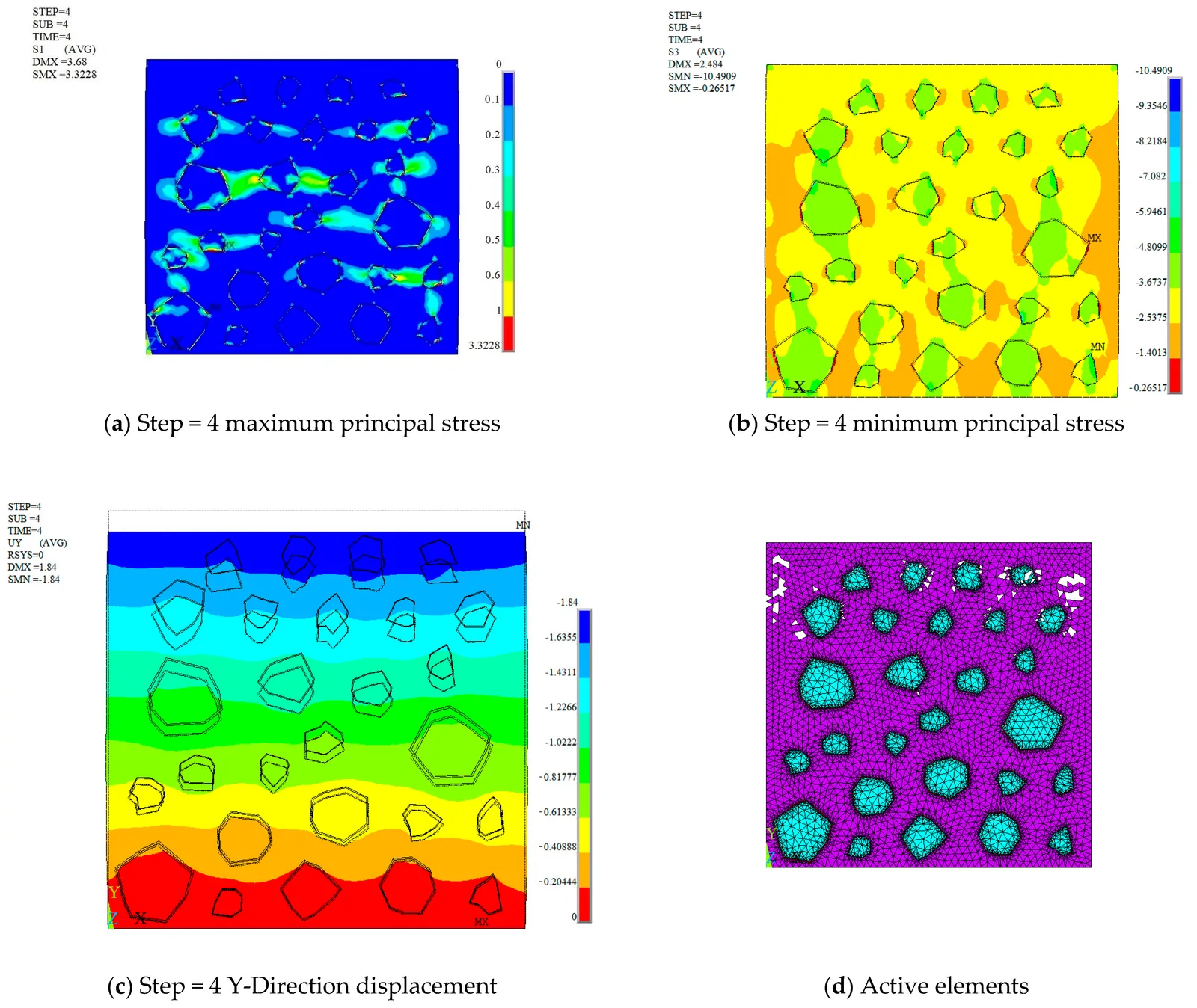
Numerical simulation process.

After completing the uniaxial compression simulations under the baseline, local perturbation, and finite-amplitude comparison cases, the compressive strength, macroscopic elastic modulus, and failure displacement were extracted from the numerical results using a consistent procedure. The macroscopic elastic modulus was defined as the secant modulus between 20% and 40% of the peak compressive stress, calculated from the stress increment divided by the corresponding strain increment within this interval. This pre-peak range was selected to characterize the effective stiffness response while reducing the influence of both the initial loading stage and pronounced damage development. The same extraction procedure was applied consistently to all simulation cases. The failure displacement was defined as the vertical displacement corresponding to the peak compressive load in the numerical load–displacement response. Because the failure displacement may depend on the adopted finite element discretization and element-deactivation treatment, it is used in this study as a comparative numerical response under identical modeling conditions rather than as a mesh-objective intrinsic material property. Representative simulation results are shown in Figure 2, and the corresponding macroscopic responses are summarized in Table 3.

For the subsequent sensitivity analysis, all macroscopic responses were extracted from the corresponding simulations using the same procedure to ensure a consistent basis for relative comparison among parameters. Compared with the baseline case, perturbations of the six mesoscopic parameters produced changes in different magnitudes and directions in the three macroscopic responses. These raw responses were then converted into dimensionless relative sensitivities to eliminate the effects of differences in units and numerical scales among the evaluation criteria.

The numerical model is primarily intended for identifying relative parameter effects and screening parameter priorities. Because the present study employs a two-dimensional random aggregate structure, linear-elastic mesoscopic constitutive relations, a maximum tensile stress failure criterion, and the element birth-and-death technique, the calculated responses should be interpreted within the adopted numerical framework. Multiple statistically independent random aggregate realizations are therefore introduced to evaluate the influence of mesostructural variability, while the geometry within each realization is kept unchanged during parameter perturbation. The remaining model-dependent limitations are further discussed in Section 4.6.

## 4. Local Sensitivity and Multi-Response Priority Analysis of Mesoscopic Parameters in CSG

### 4.1. Local Sensitivity of Mesoscopic Parameters to Macroscopic Responses

The local sensitivity coefficients of the six mesoscopic parameters were calculated using the ±5% and ±10% bidirectional perturbation results according to the central finite-difference formulation. The signed coefficient $R_{ij}$ characterizes the direction of the local response variation, whereas its absolute value $S_{ij}=|R_{ij}|$ represents the corresponding sensitivity magnitude. The results obtained under the two perturbation amplitudes are summarized in Table 4.

As shown in Table 4, the local sensitivity characteristics obtained from the ±5% and ±10% perturbations are generally consistent in both magnitude and direction. The mortar elastic modulus $E_{m}$ exhibits the highest sensitivity to the macroscopic elastic response, with sensitivity magnitudes of 0.520 and 0.530 under the ±5% and ±10% perturbations, respectively. At the same time, increasing $E_{m}$ reduces the failure displacement, with signed sensitivity coefficients of −0.270 and −0.278. This indicates that the mortar stiffness is closely associated with the overall stiffness and deformation response of the present numerical model.

The interfacial tensile strength $f_{ti}$ shows a different response pattern. Its sensitivity magnitudes to compressive strength and failure displacement reach 0.155 and 0.455 under the ±5% perturbation and increase slightly to 0.160 and 0.468 under the ±10% perturbation. In contrast, its signed sensitivity to the macroscopic elastic modulus is negative. Thus, a high sensitivity magnitude should not be interpreted as a uniformly beneficial effect of increasing the corresponding parameter; the direction of the original response variation must be considered separately.

The aggregate elastic modulus $E_{a}$ and interfacial elastic modulus $E_{i}$ exhibit intermediate sensitivity levels. The tensile strengths of the aggregate and mortar matrix, $f_{ta}$ and $f_{tm}$, produce only small changes in the three macroscopic responses within the neighborhood of the baseline state. These results indicate an evident differentiation among the six parameters under small bidirectional perturbations.

### 4.2. Stability of Sensitivity Results

To examine whether the identified parameter hierarchy depends strongly on the perturbation amplitude, the mean sensitivity magnitude over the three macroscopic responses was calculated for the ±5% and ±10% perturbations and compared with that obtained from the original +100% finite-amplitude perturbation. The comparison is presented in Table 5.

The parameter ranking remains unchanged under the three perturbation levels.


$$E_{m}>f_{ti}>E_{a}>E_{i}>f_{tm}>f_{ta}$$

The Spearman rank correlation coefficients between the ±5% and ±10% results and between the ±5% and +100% results are both 1.0000. For the four parameters with relatively pronounced sensitivities, the differences between the ±5% and ±10% mean sensitivity values are limited. Although the relative variation in the two low-sensitivity parameters is numerically larger in some cases, their absolute sensitivity values remain very small, and their priority levels do not change. These results suggest that the high-, medium-, and low-sensitivity groups are relatively stable near the baseline state, while the original +100% results can be regarded as a finite-amplitude extension of the same general response tendency rather than as the definition of local sensitivity itself.

To further examine the influence of random mesostructural configuration, the ±5% local sensitivity analysis was repeated for five statistically independent random aggregate realizations. The aggregate gradation, area fraction, material parameters, and other statistical characteristics were kept unchanged, while aggregate locations and interface distributions varied among the realizations. The statistical results are presented in Table 6.

The sensitivity magnitudes exhibit only limited dispersion among the five random realizations. More importantly, the rank ranges of all six parameters remain unchanged. $E_{m}$ and $f_{ti}$ consistently occupy the first two positions, $E_{a}$ and $E_{i}$ remain in the intermediate group, and $f_{tm}$ and $f_{ta}$ remain in the low-sensitivity group. This indicates that, within the investigated random realizations, mesostructural variability affects the numerical magnitude of the sensitivity coefficients but does not substantially alter the overall parameter hierarchy.

For the subsequent multi-response evaluation, the mean sensitivity magnitudes obtained from the five random realizations are used as the response-specific sensitivity matrix. This treatment reduces the dependence of the comprehensive ranking on a single random aggregate configuration.

### 4.3. Weights of Macroscopic Response Criteria

Compressive strength, macroscopic elastic modulus, and failure displacement were selected as evaluation criteria because they represent the load-carrying capacity, overall stiffness, and failure-deformation characteristics of the material, respectively. To determine their subjective weights, six experts with research backgrounds in CSG, hydraulic materials, mesoscopic mechanics, and numerical simulation were invited to provide independent ratings. Their relevant research or engineering experience ranged from 10 to 15 years. A 0–5 scale was adopted, where 0 denotes not important, 1–2 low importance, 3 moderate importance, 4 important, and 5 very important. All ratings were completed independently, and no conflicts of interest related to the present evaluation were declared.

The expert scores are listed in Table 7.

Based on the OWA operator and entropy weighting method, the subjective, objective, and combined weights were recalculated using the mean local sensitivity matrix in Table 6. The results are shown in Table 8.

The subjective weights remain relatively balanced among the three criteria. In contrast, the entropy-based objective weight of the macroscopic elastic modulus is the largest, reaching 0.4288, because the six mesoscopic parameters show a more pronounced differentiation in their sensitivities to this response. After combining subjective and objective information, the weights of compressive strength, macroscopic elastic modulus, and failure displacement are 0.2038, 0.4500, and 0.3463, respectively.

These weights should be interpreted as criterion importance under the present evaluation objective rather than as intrinsic properties of the material. Accordingly, the subsequent MULTIMOORA result represents a weighted multi-response parameter priority.

### 4.4. Multi-Response Parameter Priority Based on MULTIMOORA

The mean local sensitivity matrix obtained from the five random aggregate realizations was vector-normalized before the MULTIMOORA evaluation. The normalized results are given in Table 9.

The mortar elastic modulus exhibits the largest normalized sensitivity to the macroscopic elastic response, whereas the interfacial tensile strength shows the largest values for compressive strength and failure displacement. The aggregate elastic modulus and interfacial elastic modulus exhibit intermediate responses, while the two tensile strength parameters of the aggregate and mortar matrix remain relatively low.

The normalized matrix and combined weights were then introduced into the ratio system, reference point method, and modified multiplicative form. The results are presented in Table 10.

The final multi-response parameter priority is therefore:


$$E_{m}>f_{ti}>E_{a}>E_{i}>f_{tm}>f_{ta}$$

The ratio system and modified multiplicative form produce identical rankings. The reference point method maintains the same first three positions but slightly adjusts the order of $E_{i}$ and $f_{tm}$ in the lower-priority group. The Spearman rank correlation coefficient between the ratio system and the modified multiplicative form is 1.0000, while the coefficients between the reference point method and the other two methods are both 0.9429.

These results indicate that the high-priority parameter group is not dependent on a single aggregation mechanism. $E_{m}$ and $f_{ti}$ remain the two dominant candidates for subsequent parameter calibration, whereas the minor differences among the lower-priority parameters reflect the different aggregation principles of the three MULTIMOORA components.

It should be emphasized that this sequence is not interpreted as an intrinsic sensitivity order independent of the evaluation objective. Rather, it represents a multi-response parameter priority obtained by integrating response-specific local sensitivities with criterion weights.

### 4.5. Cross-Method Consistency and Weight Robustness

To examine whether the final priority is strongly dependent on the MULTIMOORA aggregation procedure, a directly weighted sensitivity was additionally calculated using the same combined weights. The results are presented in Table 11.

The directly weighted method yields exactly the same parameter order as the final MULTIMOORA result, with a Spearman rank correlation coefficient of 1.0000. Because the two methods use the same underlying sensitivity matrix and criterion weights, this agreement is interpreted only as cross-method consistency and not as independent validation of the physical accuracy of the ranking.

The dependence of the parameter priority on criterion weights was further examined using equal weighting, OWA-based subjective weighting, entropy weighting, and combined OWA–entropy weighting. The results are summarized in Table 12.

The directly weighted method produces the same ranking under all four weighting scenarios. For MULTIMOORA, $E_{m}$ and $f_{ti}$ consistently occupy the first two positions, although their internal order changes with the weighting scheme. Under equal and OWA-based subjective weighting, $f_{ti}$ ranks first, whereas $E_{m}$ becomes the highest-priority parameter under entropy and combined weighting.

This change can be attributed to the different response characteristics of the two parameters. The mortar elastic modulus exhibits a particularly high sensitivity to the macroscopic elastic response, whereas the interfacial tensile strength shows pronounced sensitivity to both compressive strength and failure displacement. Therefore, increasing the weight assigned to the macroscopic elastic modulus favors $E_{m}$, whereas a more balanced distribution of criterion weights increases the relative priority of $f_{ti}$.

The remaining parameters maintain essentially the same high-, medium-, and low-priority hierarchy under all weighting scenarios. It is therefore more appropriate to regard $E_{m}$ and $f_{ti}$ as a first-priority parameter group rather than to identify either parameter as an absolute and universally most sensitive variable.

### 4.6. Response Mechanisms and Limitations Under the Adopted Model Conditions

The sensitivity differences among the six mesoscopic parameters are associated with the roles assigned to the aggregate, mortar matrix, and interface phases in the present numerical model. The mortar matrix forms the continuous phase between adjacent aggregates and therefore directly participates in deformation compatibility and stress transfer. Across the five random aggregate realizations, the mean sensitivity of $E_{m}$ to the macroscopic elastic modulus reaches 0.5158, while its sensitivity to failure displacement is 0.2664. Moreover, the signed local response shows that increasing $E_{m}$ increases the macroscopic elastic modulus but decreases failure displacement. Thus, under the present model conditions, $E_{m}$ is mainly characterized as a high-priority stiffness-related parameter rather than as an isolated parameter governing the overall material response. 

The interfacial tensile strength $f_{ti}$ exhibits mean sensitivity magnitudes of 0.1550, 0.2039, and 0.4512 to compressive strength, macroscopic elastic modulus, and failure displacement, respectively. Because failure of the interface elements is identified using the maximum tensile stress criterion, variations in $f_{ti}$ directly affect the condition at which local interfacial failure is initiated. Accordingly, $f_{ti}$ exhibits a pronounced influence on both load-carrying and failure-deformation responses within the adopted numerical framework. This interpretation is specific to the present constitutive and failure assumptions and should not be generalized as a model-independent governing mechanism. 

The aggregate elastic modulus $E_{a}$ and interfacial elastic modulus $E_{i}$ occupy an intermediate priority level. $E_{a}$ has a relatively pronounced effect on the macroscopic elastic response, whereas $E_{i}$ mainly affects compressive strength and failure deformation under the current parameter range. In comparison, $f_{ta}$ and $f_{tm}$ exhibit relatively small local sensitivities, indicating that individual variations in these parameters produce only limited changes in the selected macroscopic responses near the baseline state. 

Several limitations should nevertheless be recognized. First, although different perturbation amplitudes are considered, the present analysis remains based on a one-at-a-time framework and therefore does not explicitly quantify interactions among mesoscopic parameters. In particular, possible interactions between mortar and interfacial properties may alter their combined effect on the macroscopic responses. Second, although five statistically independent random aggregate realizations are introduced to evaluate mesostructural variability, a finite number of realizations cannot represent all possible aggregate configurations. Finally, the two-dimensional linear-elastic model combined with the maximum tensile stress failure criterion and element birth-and-death technique provides a simplified representation of the complex damage process of actual CSG. Therefore, the results obtained in this study should primarily be interpreted as sensitivity and calibration priorities under the adopted numerical framework rather than as intrinsic physical contributions of individual constituent properties.

Overall, the perturbation-amplitude comparison, random-realization analysis, cross-method comparison, and weight-robustness assessment consistently identify $E_{m}$ and $f_{ti}$ as the first-priority parameter group. $E_{a}$ and $E_{i}$ constitute the intermediate group, while $f_{tm}$ and $f_{ta}$ have relatively low priorities. This hierarchical interpretation is more consistent with the multi-response nature of the present sensitivity assessment than assigning a universally dominant role to a single mesoscopic parameter. 

## 5. Conclusions

Based on a two-dimensional random polygonal aggregate finite element model, this study investigated the local sensitivities of the aggregate, mortar matrix, and interfacial mesoscopic parameters to the macroscopic mechanical responses of cemented sand and gravel. The stability of the sensitivity results was further examined with respect to perturbation amplitude and random mesostructural realization, and the multi-response parameter priorities were evaluated using the combined OWA–entropy weighting and MULTIMOORA methods. The main conclusions are as follows:

1. The six mesoscopic parameters exhibit distinct local sensitivities to the macroscopic responses. Across the five random aggregate realizations, the mean sensitivity of the mortar elastic modulus $E_{m}$ to the macroscopic elastic modulus and failure displacement is 0.5158 and 0.2664, respectively, while the corresponding sensitivities of the interfacial tensile strength $f_{ti}$ to compressive strength and failure displacement are 0.1550 and 0.4512. The ±5% and ±10% perturbations yield consistent sensitivity directions and parameter hierarchies, and comparison with the original +100% finite-amplitude perturbation shows that the high-, medium-, and low-sensitivity groups remain generally unchanged.
2. Under the combined OWA–entropy weighting scheme, the MULTIMOORA-based multi-response parameter priority is $E_{m}>f_{ti}>E_{a}>E_{i}>f_{tm}>f_{ta}$. The directly weighted method yields the same overall ranking, while the ratio system, reference point method, and modified multiplicative form show high cross-method consistency. The five statistically independent random aggregate realizations also preserve the same overall parameter hierarchy, indicating that the identification of the principal parameter groups is relatively stable with respect to mesostructural randomness under the investigated conditions.
3. Under equal, OWA-based subjective, entropy-based objective, and combined weighting schemes, $E_{m}$ and $f_{ti}$ consistently remain the two highest-priority parameters, whereas $E_{a}$ and $E_{i}$ form an intermediate priority group and $f_{tm}$ and $f_{ta}$ remain relatively low-priority parameters. The internal order of $E_{m}$ and $f_{ti}$ varies with the criterion weights, indicating that the final ranking should be interpreted as a model- and evaluation-objective-dependent parameter priority rather than as an intrinsic material sensitivity order. The proposed framework therefore provides a quantitative basis for mesoscopic parameter screening and subsequent calibration of CSG numerical models.

## Figures and Tables

**Table 1:** Baseline equivalent mesoscopic parameters.

Parameter	Baseline Value
$E_{a}$	500 MPa
$E_{m}$	100 MPa
$E_{i}$	40 MPa
$f_{ta}$	5 MPa
$f_{tm}$	1 MPa
$f_{ti}$	0.5 MPa

**Table 2:** Multilevel perturbation scheme for mesoscopic parameters.

Perturbation Level	Relative Parameter Value	Purpose
−10%	0.90$x_{i0}$	Local sensitivity stability
−5%	0.95$x_{i0}$	Local sensitivity
Baseline	$x_{i0}$	Reference state
+5%	1.05$x_{i0}$	Local sensitivity
+10%	1.10$x_{i0}$	Local sensitivity stability
+100%	2.00$x_{i0}$	Finite-amplitude comparison

**Table 3:** Numerical responses of CSG material.

Group	Compressive Strength/MPa	Elastic Modulus/MPa	Failure Displacement/mm
A1	6.83	238.24	3.02
A2	7.01	271.43	2.72
A3	7.46	370.94	2.12
A4	7.48	237.13	3.32
A5	6.8	237.07	3.02
A6	6.91	241.1	3.02
A7	7.94	184.99	4.52

**Table 4:** Local sensitivities of mesoscopic parameters under ±5% and ±10% perturbations.

Mesoscopic Parameter	Compressive Strength		Elastic Modulus		Failure Displacement	
	$R^{5\%}$	$R^{10\%}$	$R^{5\%}$	$R^{10\%}$	$R^{5\%}$	$R^{10\%}$
$E_{a}$	+0.035	+0.036	+0.155	+0.158	−0.095	−0.097
$E_{m}$	+0.085	+0.088	+0.520	+0.530	−0.270	−0.278
$E_{i}$	+0.090	+0.093	−0.006	−0.006	+0.085	+0.088
$f_{ta}$	−0.005	+0.006	−0.004	−0.005	+0.001	+0.001
$f_{tm}$	+0.012	+0.013	+0.010	+0.011	+0.002	+0.002
$f_{ti}$	+0.155	+0.160	−0.205	−0.213	+0.455	+0.468

**Table 5:** Comparison of sensitivity levels under different perturbation amplitudes.

Parameter	±5% Mean Sensitivity	Rank	±10% Mean Sensitivity	Rank	+100% Finite-Amplitude Sensitivity	Rank
$E_{a}$	0.0950	3	0.0970	3	0.0883	3
$E_{m}$	0.2917	1	0.2987	1	0.3157	1
$E_{i}$	0.0603	4	0.0623	4	0.0664	4
$f_{ta}$	0.0033	6	0.0040	6	0.0031	6
$f_{tm}$	0.0080	5	0.0087	5	0.0079	5
$f_{ti}$	0.2717	2	0.2803	2	0.2942	2

**Table 6:** Local sensitivity statistics for five random aggregate realizations.

Parameter	Compressive Strength	Elastic Modulus	Failure Displacement	Mean Comprehensive Sensitivity	Rank Range
$E_{a}$	0.0348 ± 0.0009	0.1512 ± 0.0045	0.0949 ± 0.0022	0.0936	3–3
$E_{m}$	0.0864 ± 0.0013	0.5158 ± 0.0218	0.2664 ± 0.0087	0.2895	1–1
$E_{i}$	0.0900 ± 0.0015	0.0058 ± 0.0002	0.0845 ± 0.0025	0.0601	4–4
$f_{ta}$	0.0050 ± 0.0003	0.0040 ± 0.0002	0.0010 ± 0.0001	0.0033	6–6
$f_{tm}$	0.0124 ± 0.0015	0.0105 ± 0.0011	0.0020 ± 0.0002	0.0083	5–5
$f_{ti}$	0.1550 ± 0.0057	0.2039 ± 0.0072	0.4512 ± 0.0100	0.2700	2–2

**Table 7:** Expert ratings of macroscopic response criteria.

Criterion	Expert 1	Expert 2	Expert 3	Expert 4	Expert 5	Expert 6
Compressive strength	4.3	3.1	3.5	1.7	2.9	2.6
Elastic modulus	3.6	2.6	2.9	3.9	3	4.2
Failure displacement	2.9	4.4	1.9	2.8	4.5	2.7

**Table 8:** OWA–entropy weighting results.

Criterion	OWA Aggregate Score	Subjective Weight/wˢ	Entropy/e	Objective Weight/wᵒ	Combined Weight/w
Compressive strength	3.0156	0.3193	0.7965	0.2149	0.2038
Elastic modulus	3.3375	0.3534	0.5941	0.4288	0.4500
Failure displacement	3.0906	0.3273	0.6627	0.3563	0.3463

**Table 9:** Vector-normalized local sensitivity matrix.

Mesoscopic Parameter	Compressive Strength	Macroscopic Elastic Modulus	Failure Displacement
$E_{a}$	0.1721	0.2629	0.1761
$E_{m}$	0.4269	0.8970	0.4942
$E_{i}$	0.4446	0.0101	0.1567
$f_{ta}$	0.0245	0.0070	0.0019
$f_{tm}$	0.0613	0.0182	0.0038
$f_{ti}$	0.7655	0.3547	0.8368

**Table 10:** MULTIMOORA results under combined OWA–entropy weights.

Parameter	Ratio System Value/Rank	Reference Point Deviation/Rank	Multiplicative Value/Rank	Final Rank
$E_{a}$	0.2143/3	0.2853/3	1.2135/3	3
$E_{m}$	0.6617/1	0.1186/1	1.6480/1	1
$E_{i}$	0.1494/4	0.3991/5	1.1387/4	4
$f_{ta}$	0.0088/6	0.4004/6	1.0088/6	6
$f_{tm}$	0.0220/5	0.3954/4	1.0218/5	5
$f_{ti}$	0.6054/2	0.2440/2	1.5888/2	2

**Table 11:** Directly weighted sensitivity and ranking consistency.

Mesoscopic Parameter	Directly Weighted Sensitivity/$C_{i}$	Final MULTIMOORA Rank
$E_{m}$	0.3419	1
$f_{ti}$	0.2796	2
$E_{a}$	0.1080	3
$E_{i}$	0.0502	4
$f_{tm}$	0.0080	5
$f_{ta}$	0.0032	6

**Table 12:** Parameter priorities under different weighting scenarios.

Weighting Scheme	Compressive Strength	Elastic Modulus	Failure Displacement	Directly Weighted Ranking	MULTIMOORA Ranking
Equal weighting	0.3333	0.3333	0.3333	$E_{m}>f_{ti}>E_{a}>E_{i}>f_{tm}>f_{ta}$	$f_{ti}>E_{m}>E_{a}>E_{i}>f_{tm}>f_{ta}$
OWA	0.3193	0.3534	0.3273	$E_{m}>f_{ti}>E_{a}>E_{i}>f_{tm}>f_{ta}$	$f_{ti}>E_{m}>E_{a}>E_{i}>f_{tm}>f_{ta}$
Entropy weighting	0.2149	0.4288	0.3563	$E_{m}>f_{ti}>E_{a}>E_{i}>f_{tm}>f_{ta}$	$E_{m}>f_{ti}>E_{a}>E_{i}>f_{tm}>f_{ta}$
Combined weighting	0.2038	0.4500	0.3463	$E_{m}>f_{ti}>E_{a}>E_{i}>f_{tm}>f_{ta}$	$E_{m}>f_{ti}>E_{a}>E_{i}>f_{tm}>f_{ta}$

## Data Availability

The original contributions presented in this study are included in the article. Further inquiries can be directed to the corresponding author.

## References

[1] Guo L., Zhang Y., Zhong L., Zhang F., Wang M., Li S. (2020). CSG Elastic Modulus Model Prediction Considering Meso-components and its Effect. Sci. Eng. Compos. Mater..

[2] Huang X. (2021). Analysis of Macro–Mesoscopic Parameters of Sand Based on Random Forest and the Discrete Element Method. Master’s Thesis.

[3] Li K.Y., Yang G.Y., Li L.J., Xu Y.L. (2020). Analysis of the relevance between macro-micro parameters for clays based on particle flow simulation. J. Exp. Mech..

[4] Xu J.M., Huang D.Y., Zhu H.C. (2016). Relations between macro- and meso-scopic mechanical parametersof granite based on actual distributions of mesocompositions. Chin. J. Rock Mech. Eng..

[5] Huang H., Li P., Huo W.L., Zhang X.C. (2020). Study on Relationship between Mesoscopic and Macroscopic Mechanical Parameters and Failure Model of Cemented Sand and Gravel Material. J. N. China Univ. Water Resour. Electr. Power (Nat. Sci. Ed.).

[6] Benvenuti L., Kloss C., Pirker S. (2016). Identification of DEM simulation parameters by Artificial Neural Networks and bulk experiments. Powder Technol..

[7] Matin S.S., Farahzadi L., Makaremi S., Chelgani S.C., Sattari G. (2018). Variable selection and prediction of uniaxial compressive strength and modulus of elasticity by Random Forest. Appl. Soft Comput..

[8] Zhang W., Wu C., Li Y., Wang L., Samui P. (2019). Assessment of pile drivability using random forest regression and multivariate adaptive regression splines. Georisk Assess. Manag. Risk Eng. Syst. Geohazards.

[9] Puri N., Prasad H.D., Jain A. (2018). Prediction of Geotechnical Parameters Using Machine Learning Techniques. Procedia Comput. Sci..

[10] Pham B.T., Qi C., Ho L.S., Nguyen-Thoi T., Al-Ansari N., Nguyen M.D., Nguyen H.D., Ly H.-B., Van Le H., Prakash I. (2020). A Novel Hybrid Soft Computing Model Using Random Forest and Particle Swarm Optimization for Estimation of Undrained Shear Strength of Soil. Sustainability.

[11] Park S., Kim J. (2019). Landslide Susceptibility Mapping Based on Random Forest and Boosted Regression Tree Models, and a Comparison of Their Performance. Appl. Sci..

[12] Wang Y., Liu Z. (2026). Assessing the Sustainable Synergy Between Digitalization and Decarbonization in the Coal Power Industry: A Fuzzy DEMATEL-MultiMOORA-Borda Framework. Sustainability.

[13] Liu J.Y., Zhang K., Wang G.H. (2018). Comparative study of data standardization methods in comprehensive evaluation. Digit. Technol. Appl..

[14] Yage R.R.R. (1993). Families of OWA operators. Fuzzy Sets Syst..

[15] Guan G., Li W.W., Han H.K. (2019). Performance evaluation of urban sewage treatment based on the entropy-weight TOPSIS method. Yangtze River.

[16] Saranya M., Jayanthi D. (2025). Cubic Fermatean Fuzzy MEREC–MULTIMOORA Approach for Healthcare Waste Management System. Int. J. Fuzzy Syst..

[17] Guo L., Zhang Y., Zhong L., Wang M., Zhu X. (2020). Study on Macroscopic and Mesoscopic Mechanical Behavior of CSG based on Inversion of Mesoscopic Material Parameters. Sci. Eng. Compos. Mater..

[18] Zhang Y. (2021). Multiscale Study on the Parameters of the Constitutive Model for Cemented Sand and Gravel Materials. Master’s Thesis.

[19] Bažant Z.P., Ozbolt J. (1990). Nonlocal microplane model for fracture damage and size effect in structures. J. Eng. Mech. ASCE.

[20] Zhu W.C., Teng J.G., Tang C.A. (2002). Numerical simulation of strength envelope and fracture patterns of concrete under biaxial loading. Mag. Concr. Res..

[21] Yuan Z.X. (2019). Correlation Analysis Between the Mesostructure and Mechanical Characteristics of Concrete Based on a Random Aggregate Model. Ph.D. Thesis.

[22] Comi C., Perego U. (2001). Fracture energy based bi-dissipative damage model for concrete. Int. J. Solids Struct..

